# Stress distribution and displacement in maxillary anterior teeth during simultaneous intrusion and retraction using a 3D FEA model

**DOI:** 10.6026/973206300222409

**Published:** 2026-04-30

**Authors:** Syed Abid Hussain, Syed Zameer Khurshaid, Reyazahmad Ganie, Irfan Bashir, G. Dinesh Kumar

**Affiliations:** 1 Department of Orthodontics and Dentofacial orthopaedics, Government Dental College, Srinagar, Jammu & Kashmir, India

**Keywords:** Finite element analysis (FEA), K-SIR arch, orthodontic tooth movement, three-piece intrusion arch, stress distribution, intrusion, Retraction

## Abstract

Finite Element Analysis (FEA) helps to visualize stress, strain and displacement and biomechanics of a system. Therefore, it is of interest to assess the effects of various methods on simultaneous intrusion and retraction of maxillary anterior teeth by Three-dimensional FEA. For this investigation, the 3D Computer Aided Design (CAD) models simulating Maxilla, three-piece intrusion arch, K-SIR arch and TADs were modeled in the computer finite element program. The software used for geometric modelling was Autodesk AutoCAD 2017 and Inventor 2017. The von Mises stress, principal compressive and tensile stress on PDL and alveolar bone and initial displacement of the teeth in bucco- palatal, mesio- distal and vertical direction were analyzed. Thus, the three-piece intrusion arch showed better stress distribution and controlled tooth movement compared to K-SIR arch and TADs model.

## Background:

Concept of FEM originated during 1940s to study stresses in complex airframe structures. Mathematical foundation was laid down during 1940s and 1950s. CLOUGH coined the term 'Finite Element' in 1960. Finite Element Analysis was developed first by R. Courant in 1943. The first dental FEA was executed by Farah *et al.* in 1973. In orthodontics, FEA was introduced by Yettram *et al.* in (1977) [1]. Finite Element Analysis (FEA) is a computer based numerical method to calculate the strength and behaviour of materials. It is a technique for obtaining approximate numerical solutions to abstract equations of calculus used to predict the response of physical systems that are subjected to external influences. A complex structure is "meshed" or subdivided into many building blocks, called finite elements, which are then easier to deal with mathematically [2]. In the general field of medicine, FEM has been applied mainly to orthopedic research in which the mechanical responses of bony structures relative to external forces were studied. Furthermore, some research has been carried out to investigate soft-tissue and skeletal responses to mechanical forces. In orthodontics, FEM has been used to elucubrate the effect of forces on teeth, periodontal ligament (PDL), bone and orthodontic appliances. Orthodontics can shape and analyse any material or dentomaxillofacial structures by applying the FEM [3]. Around the world, dental protrusion with deep overbite is common in many ethnic groups. The orthodontic correction being associated with a troublesome biomechanical challenge, asuprighting of incisors often lengthens the crown vertically and increases the overbite.

Three-dimensional (3D) control during retraction of upper anterior teeth is fundamental for facial esthetics, appropriate functioning of the stomatognathic system and stability of orthodontic treatment. Hence, synchronous intrusion and retraction of the anterior teeth may be desirable to accomplish the optimum treatment results (Shroff *et al.* 1995, 1997) [4]. In clinical practice, the simultaneous intrusion and retraction of the maxillary anterior teeth can be carried out using various mechanics. The Three-piece intrusion arch was introduced in 1995 by Shroff, Lindauer, Burstone and Leiss for the purpose of simultaneous intrusion and retraction of flared anterior teeth. Also, for the correction of their axial inclinations with better anchorage control [5]. The K-SIR is the modified arch wire based on Burstone and Nanda's segmented loop mechanics [6]. K-SIR arch wire can be used for en masse retraction of the six anterior teeth in first premolar extraction cases that require maximum anchorage as well as intrusion for correction of deepbite [5]. The TADs have replaced many traditional mechanics and simplified orthodontic treatment. These days, the miniplates also have been considerably used in situations and are quite popular due to added stability they exhibit in comparison to TADs [6, 7]. However, mini screws have various advantages as they are cost effective, can be placed and removed easily and are small. Thus, mini screws can be implanted comfortably in most sites and thus are the most popular absolute support today [8]. Therefore, it is of interest to evaluate and compare the stress distribution and displacement of maxillary anterior teeth during simultaneous intrusion and retraction using various methods, by 3D finite element analysis.

## Materials and Methods:

This FEM Study was carried out at a specialized centre, AnaMac Design 301-3rd Floor Good Earth City Center Sec 50 Gurgaon Delhi. The details of each step of all the procedures carried out in this study have been mentioned below.

Three of the mechanics that can be used for simultaneous intrusion and retraction of anterior teeth are:-

[1] Three-piece intrusion arch.

[2] Kalra simultaneous intrusion and retraction arch (K-SIR).

[3] Temporary anchorage device assisted simultaneous intrusion and retraction of maxillary anterior teeth.

## Inclusion criteria:

Detailed drawings of a model of maxilla and maxillary dentition without first premolars, Mini-implants: 8 x1.5mm, Mini-implant height 6mm from the CEJ between second premolar and first molar on buccal surface, Nickel Titanium (NiTi) Closed Coil Spring: length 9mm, arbor diameter 0.03", Preadjusted Edgewise Appliance (PEA) 0.022" x 0.028" MBT brackets and Arch wires: 0.019" x 0.025" stainless steel wire, 0.019" x 0.025" TMA wire, 0.017" x 0.025" TMA wire, 0.021" x 0.025" stainless steel wire.

## Exclusion criteria:

Mini-implant dimensions other than 8 x 1.5mm, Mini-implant placement site other than at a height 6mm from the CEJ between second premolar and first molar on buccal surface, NiTi Closed Coil Spring length other than 9mm and arbor diameter other than 0.03", Arch wires other than 0.019" x 0.025" stainless steel wire, 0.019" x 0.025" TMA wire, 0.017" x 0.025" TMA wire, 0.021" x 0.025" stainless steel wire and PEA other than 0.022" x 0.028" MBT brackets. 3D Computer-Aided Design (CAD) models simulating the maxilla, three-piece intrusion arch, K-SIR arch, TADs were modelled in the computerized finite element program as per the detailed drawings produced.

## Method of collection of data:

## CAD modeling:

3D CAD model of maxilla will be modelled as per the detailed drawings provided. The software used for the geometric modeling was AUTOCAD 2017, INVENTOR 2017.

## Pre-processing (Meshing):

These geometric models were converted into the finite element models. The finite element modeling was the representative of the geometry in terms of finite elements and nodes. This processing is called as discretization. These elements are considered interconnected at joints which are called as nodes. In this study the finite element nodal generation was done according to the HYPERMESH 2019. The number of nodes and elements used for different models are depicted in Table 1 (see PDF). Various structures involved in this study include the cancellous bone, cortical bone, PDL, teeth and different wire components and titanium mini-implants. Respective material properties were assigned [9] (Table 2 - see PDF).

## Boundary conditions and loads:

To prevent the models from free body motion, the boundary conditions were defined to avoid free movement of the tooth. All the materials used in the three models were assumed to be homogenous, isotropic and of linear elasticity. The traction forces were applied to the various models.

## Finite element solution:

Finite element models were solved for structural analysis and the assessment of the stress and displacements were performed using ANSYS 2019 software.

## Evaluation parameters:

To evaluate the stress distribution and displacement of maxillary anterior teeth using Three-piece intrusion arch, K-SIR arch and in TADs model. To compares the obtained stress distribution values and displacements with each other.

## Results:

After the construction of the 3D geometric models and after the application of loads, the results were obtained. The result of the analysis is called (post processing). Stresses (MPa) and displacements (millimetre (mm)) were calculated and represented in colourful bands, where different colours represent different stress levels and different displacements. Red colour of the spectrum indicates maximum stress/displacement and blue representing minimum stress/displacement. The constructed models were imported into ANSYS software for analysing the displacement and stress distribution under the influence of retractive and intrusive forces. After evaluating the initial tooth movement in all the three models, the results were as follows: the intrusion of anterior teeth in the vertical plane was noted in all the three models with maximum in three-piece intrusion arch and minimum in the TADs model (Table 3 - see PDF). In the sagittal plane maximum displacements were seen in the TADs model with bodily tooth movement in three-piece intrusion arch and tipping in other two models. The canine showed tipping, which were maximum in the TADs model, molars showed tipping tooth movement in three piece and K-SIR arch model. There was slight extrusion of molars in three-piece intrusion arch and TADs model with anchor loss, maximum in three-piece intrusion arch followed by K-SIR arch model. The principle compressive stress, principle tensile stress and Von Mises stress were maximum in the cortical bone and minimum in the PDL for all the three models. The maximum principle compressive stress was found in the region of the canine in cortical bone. The maximum principle tensile stress was found in the region of lateral incisor in cortical bone. The maximum von Mises stress was found in the region of the lateral incisor and canine in cortical bone. In all the three models, maximum von Mises stress in the PDL was found in the cervical area and minimum in the apical area (Table 4 - see PDF).

## Discussion:

Since uprighting incisors frequently lengthens the crown vertically and increases the overbite, orthodontic treatment is accompanied with a challenging biomechanical issue. Dental protrusion with deep overbite is widespread in many ethnic groups worldwide [4]. To get the best treatment outcomes, it can be preferable to simultaneously intrude and retract the anterior teeth. During intrusion of the anterior teeth, control of their labio-lingual axial inclinations is critical for successful completion of treatment [10]. The effects of geometrical and material alterations under load and internal mechanical processes can be studied using FEM, a frequently used experimental research technique. Mesh convergence was used in the current study to improve the accuracy of the results and the finite element models were derived from the precise drawing created. Many studies have evaluated stress in the underlying alveolar bone [11, 12] and PDL with varying heights of implants and retraction hooks [13] for en masse retraction. But no study has evaluated the stress distribution and initial displacements comparing three-piece intrusion arch and K-SIR arch and temporary anchorage devices for simultaneous intrusion and retraction [4]. This study was performed to compare the stress distribution and displacement in central incisor, lateral incisor, canine and first molar using three methods of simultaneous intrusion and retraction. The three-piece intrusion arch, K-SIR arch and temporary anchorage devices for simultaneous intrusion and retraction were compared in the current study using FEA. Three types of stress: principle compressive stress, principle tensile stress and von Mises stress were evaluated in the cancellous bone, cortical bone and PDL. Tissue reaction to orthodontic tooth movement is known to occur either through bone or with bone. In order to comprehend tooth movement, it is crucial to investigate tensions in the alveolar bone [11]. Stresses in cortical bone were higher in this investigation than in cancellous bone, which is consistent with earlier findings by Namburi *et al.* [14] and Kole [15]. The principle compressive stress in the cortical bone is maximum in three-piece intrusion arch model and minimum in K-SIR arch model. The principle compressive stress in the cancellous bone is maximum in TADs model and minimum in K-SIR arch model. The principle compressive stress in the PDL is maximum in the TADs model and negligible in three-piece intrusion arch model. The maximum principle compressive stress was found in the region of the canine in cortical bone [16]. The principle tensile stress in the cortical bone is maximum in the TADs model and minimum in K-SIR arch model. Tensile stress in the cancellous bone is maximum in the TADs model and minimum in the K-SIR arch model. The principle tensile stress in the PDL is maximum in the three-piece intrusion arch model and minimum in K-SIR arch model. The maximum principle tensile stress was found in the region of lateral incisor in cortical bone. These results were in accordance with the results found by Field *et al.* [16], Heravi *et al.* [17] and Panika *et al.* [18]. The von Mises stress is maximum in the cortical bone and minimum in the PDL. In the cortical bone, maximum von Mises stress was found in the TADs model and minimum in the K-SIR arch model. The von Mises stress in the cancellous bone was found maximum in the TADs model and minimum in the K-SIR arch model. In the PDL, maximum von Mises stress was found in the TADs model and minimum in the K-SIR arch model. The maximum von Mises stress was found in the region of the lateral incisor and canine in cortical bone. Because of their short roots, the lateral incisor region experienced the highest stresses in the current investigation. According to Vecilli and Burstone [19], when the same load is applied, the stress magnitudes in the PDL for larger teeth are smaller and greater for smaller teeth because larger teeth have more PDL and root support. The second area of focus in the present study was the displacement. The displacement of the maxillary four incisors, canine and first molars were calculated for all the three groups. The overall displacement was observed in all three planes of space. In the transverse plane, the displacement of the teeth was constant in all the groups. In the sagittal plane, there occurred bodily movement of central incisors in three-piece intrusion arch and K-SIR arch while there was tipping of central incisor in the TADs model. These results were in accordance with that of Ahuja *et al.* [4], Bohra *et al.* [20], Namburi *et al.* [14] and Maheshwari *et al.* [21]. In case of lateral incisors, same tooth movements were seen as that of the central incisor in all the three models. For the four anterior teeth in the three-piece intrusion arch, the force vector was delivered at the CRES11, resulting in controlled tooth movement. Clockwise moments and palatal tipping of the incisors were caused by the force vector in the K-SIR arch being distal to the CRES of six anterior teeth [22]. While in case of the TADs model, the force passed more distal to the centre of resistance of anterior teeth. To redirect the line of force passing through the centre of resistance, either the height for placement of implant or the length of the power arm is to be altered [14]. The vertical intrusion of the maxillary incisors was calculated in all the three groups. Maximum intrusion was found to occur in the three-piece intrusion arch model fallowed by K-SIR arch model and least in the TADs model. Same results were found related to the canine tooth. However, in case of molar tooth there was no movement in the vertical direction in the K-SIR model and there was extrusion of molar in the molar in TADs model. The reason being that in the TADs model there was a continuous arch present as a result when intrusive forces were applied on the anterior teeth, reactionary forces brought about extrusion in the molar region. So, we can say that the deep bite in case of TADs model reduced because of incisor intrusion as well as molar extrusion. These results are in accordance with the studies carried out by Ahuja *et al.* [4] Namburi *et al.* [14] Bohra *et al.* [20] and Gupta *et al.* [23].

## Limitation of the present study:

Since the final outcomes of a FEM heavily rely on the models created, they must be created in a way that makes them comparable to actual objects in several ways. This is an in vitro study and the outcome cannot be directly extrapolated to clinical conditions. Despite their anisotropy and non-linear behavior, the periodontal ligament, tooth and alveolar bone were regarded as isotropic structures in this study. The inclination of the maxillary incisors at the start of treatment is another important key factor to measure, that can affect the point and direction of force and the intrusion and retraction attained. This was not incorporated in the present study. Another constraint of the study would be the inability to directly forecast long-term tooth movement quantitatively through simulation. As FEM can only calculate the initial tooth displacement (1 second) and stress distribution following force application.

## Conclusion:

Body movement was observed in three-piece intrusion arch and tipping in K-SIR arch and TADs model with more tipping in the TADs model. It was found that the cortical bone had the highest level of stress and that the laterals had more stresses than the centrals. The principle compressive stress and von Mises stress was highest in the TADs model followed by three-piece intrusion arch and then by K-SIR arch. Thus, the three-piece incursion arch outperformed the K-SIR arch and TADs model in terms of stress distribution and controlled tooth movement.

## Advancement to knowledge:

By enabling non-invasive, 3D simulation of orthodontic forces and exposing accurate stress distributions in the PDL and bone, FEA has greatly increased our understanding of maxillary anterior tooth biomechanics. Important developments include the assessment of anchoring loss. Especially in mini screw-assisted procedures, the quantification of tooth displacement (such as tipping and translation) and the optimization of retraction and intrusion mechanics.
